# Jasmonic acid-related resistance in tomato mediates interactions between whitefly and whitefly-transmitted virus

**DOI:** 10.1038/s41598-017-00692-w

**Published:** 2017-04-03

**Authors:** Yan-Chun Sun, Li-Long Pan, Feng-Ze Ying, Ping Li, Xiao-Wei Wang, Shu-Sheng Liu

**Affiliations:** Ministry of Agriculture Key Laboratory of Agricultural Entomology, Institute of Insect Sciences, Zhejiang University, Hangzhou, 310058 China

## Abstract

The indirect interactions between insect vectors, such as whiteflies, and the viruses they transmit, such as begomoviruses, via host plants may produce a range of outcome depending on the species/strain of each of the three organisms involved, and the mechanisms underlying the variations are not well understood. Here, we observed the performance of whiteflies on three types of tomato, which vary in level of jasmonic acid (JA)-related resistance and were either uninfected or infected by a begomovirus, to investigate the role of JA-related resistance in mediating whitefly-begomovirus interactions. Compared to the performance of whiteflies on plants of the wild type, the performance was elevated on plants deficient in JA-related resistance but reduced on plants with a high level of JA-related resistance. Further, on plants with a high level of JA-related resistance, the whitefly performed better on virus-infected than on uninfected plants; however, on tomato plants deficient in JA-related resistance, whitefly performance was less affected by the virus-infection of plants. Additionally, the expression of the JA-regulated defense gene *PI-II* in tomato plants was repressed by virus infection. These findings suggest that JA-related resistance plays an important role in the tripartite interactions between whitefly, begomovirus and tomato plant.

## Introduction

In recent decades, increasing attention has been given to the research on the tripartite interactions between insect vectors, viruses and plants^1, 2^. Many case studies have shown that the effects of the tripartite interactions may constitute major determinants of the population dynamics of the organisms in the field^3, 4^. Both direct and indirect interactions between the three organisms may operate in a complex network resulting in various consequences. For example, the indirect interactions between insect vectors and the viruses they transmit via their host plants may produce positive, neutral to negative effects on the performance of the insect vectors and in turn on the transmission of the viruses and the performance of the plants^2, 4–6^.

The whitefly *Bemisia tabaci* (Gennadius) (Hemiptera: Aleyrodidae) is a species complex composed of at least 35 morphologically indistinguishable cryptic species, some of which are invasive and important crop pests worldwide^7–11^. As the vectors of begomoviruses, which are the largest and economically the most important group of plant viruses in tropical and sub-tropical regions, the outbreaks of whiteflies in many regions are often accompanied by epidemics of plant diseases caused by begomoviruses, such as the virus diseases of tomato and other crops caused by Tomato yellow leaf curl virus (TYLCV) that have spread around the world in the past three decades^12–16^.

Like in other systems that have been investigated, plant-mediated whitefly-begomovirus interactions exert important influences on the population dynamics of the whiteflies and the epidemiology of the virus diseases^17–19^. These interactions are often complicated. For example, the effects of virus infection of the host plants on whitefly performance can be positive, negative or neutral depending on the combinations of whiteflies, begomoviruses and plants^6, 17, 18^. Thus, in some cases, a begomovirus may cause infected plants to promote the performance of its transmitting vector, which accelerates the spread of the pathogen, while in other cases the infection of the host plants by a begomovirus may have no or negative effects on the performance of the whiteflies^6, 20, 21^. The mechanisms underlying these variations remain largely unknown.

In this study, we firstly observed the performance of two cryptic species of whiteflies on three types of tomato plants that are known to vary in the level of jasmonic acid (JA)-related resistance. Next, we compared the performance of whiteflies on uninfected and begomovirus-infected plants for each type of the tomato. Finally, we examined the relative expression levels of genes related to JA signaling pathway between uninfected and virus-infected tomato plants. Our objectives were to investigate whether JA-related resistance participates in the tripartite interactions between whiteflies, begomoviruses and tomato plants.

## Materials and Methods

### Whitefly, virus and plant

The MEAM1 whitefly (*mtCO1* GenBank accession no: KM821540) and MED whitefly (*mtCO1* GenBank accession no: GQ371165) of the *B. tabaci* species complex were reared on cotton *Gossypium hirsutum* L. cv. Zhemian 1793 in insect-proof cages at 27 ± 1 °C, 70 ± 10% RH and a 14 L:10D light cycle in climate-controlled rooms. The purity of the cultures was monitored every three generations using PCR-RFLP (PCR-restriction fragment length polymorphism) and *mtCO1* sequencing analysis as described before^22^.

Clones of TYLCV isolate SH2 (GenBank Acc. No.:AM282874) were provided by Professor Xueping Zhou, Institute of Biotechnology, Zhejiang University.

For plants of tomato S*olanum lycopersicum*, the cv. *CastleMart* was used as the wild type, and JA-deficient *spr2* mutant plants and JA-overexpression *35S-prosystemin* transgenic plants, both in the *CastleMart* background, were used. To obtain virus-infected tomato plants, each of the test plants at the 3–4 true-leaf stage was inoculated with 0.2 ml of the TYLCV culture by agroinoculation as previously described^23^. All virus-inoculated and un-inoculated plants were grown in a greenhouse under natural lighting supplemented with artificial illumination (light 05:00–18:00 hours) and controlled temperature at 25 ± 3 °C, and were used for whitefly bioassays when they reached the 6–7 true-leaf stage (approximately 25 days after agroinoculation of the virus). Virus infection of plants was determined by typical disease symptoms and further conformed by PCR^24^.

### Performance of whiteflies on the three types of tomato plants

We designed six treatments, using two species of whiteflies MEAM1 and MED and three types of tomato *CastleMart*, *spr2* and *35S-prosystemin*, to investigate the effect of JA-regulated defence of the plants to whiteflies (Table 1). We observed the adult survival and fecundity of each species of whitefly feeding on each of the three types of tomato plants. For each treatment, 10–19 replicates were conducted, and for each replicate, newly emerged whiteflies (10 females and 10 males) were collected from the cultures on cotton and released onto the lower surface of a plant leaf (third to fourth leaf from the top) enclosed in a clip cage to feed, mate and oviposit. Seven days later, the live adult whiteflies in each replicate were recorded, and the eggs deposited on the leaf were counted under a microscope.

**Table 1:** Performance of MEAM1 and MED whiteflies reared on plants of tomato cv*. CastleMart, spr2 and 35S-prosystemin*.

Whitefly	Plant	No. of repl.	Survival of females % ± SEª	Survival of males % ± SE^b^	No. of eggs per female ± SE^c^
MEAM1	*CastleMart*	13	77.7 ± 3.9^b^	72.3 ± 4.7^a^	20.5 ± 1.6^c^
	*spr2*	17	88.8 ± 1.9^a^	73.5 ± 3.5^a^	30.5 ± 1.3^b^
	*35S-prosystemin*	10	42.0 ± 6.3^c^	63.0 ± 5.2^b^	20.7 ± 2.1^c^
MED	*CastleMart*	16	76.9 ± 4.6^b^	68.1 ± 5.2^a^	27.0 ± 2.1^b^
	*spr2*	19	80.5 ± 3.8^a,b^	71.1 ± 6.2^a^	55.3 ± 2.6^a^
	*35S-prosystemin*	11	26.4 ± 4.1^c^	45.5 ± 5.9^b^	19.1 ± 2.0^c^

Live adults and eggs were counted seven days after the newly emerged whitefly adults were released onto the plants to assess adult survival and fecundity.

ªTwo-factor ANOVA: *F*_whitefly_ = 2.920, *df* = 1, p > 0.05; *F*_plant_ = 49.005, *df* = 2, p < 0.05; *F*_whitefly*plant_ = 1.225, *df* = 2, p > 0.05.

^b^Two-factor ANOVA: *F*_whitefly_ = 1.657, *df* = 1, p > 0.05; *F*_plant_ = 5.477, *df* = 2, p < 0.05; *F*_ctyptic*plant_ = 1.074, df = 2, p > 0.05.

^c^Two-factor ANOVA: *F*_whitefly_ = 31.519, *df* = 1, p < 0.05; *F*_plant_ = 72.987, *df* = 2, p < 0.05; *F*_whitefly*plant_ = 21.071, *df* = 2, p < 0.05.

Different lett**e**rs in the same column indicate significant differences at P ≤ 0.05 level.

### Performance of whiteflies on uninfected and TYLCV-infected tomato plants

Due to the workload and space required for the bioassays, we tested each of the three types of tomato plants at a time. For each of the three types of plants, i.e. *CastleMart*, *spr2* and *35S-prosystemin*, we designed four treatments involving two species of whiteflies MEAM1 and MED and two levels of status of the plants, i.e. uninfected and TYLCV-infected, to observe the effects of TYLCV infection of the plants on whitefly performance (Tables 2–4). For each treatment, we conducted 10–19 replicates. The protocols for observing whitefly adult survival and fecundity were the same as above.

**Table 2:** Performance of MEAM1 and MED whiteflies reared on uninfected and TYLCV-infected plants of tomato cv. *CastleMart*.

Whitefly	Status of plants	No. of repl.	Survival of females % ± SEª	Survival of males % ± SE^b^	No. of eggs per female ± SE^c^
MEAM1	Uninfected	15	58.0 ± 5.8^b^	60.7 ± 6.4^a,b^	25.3 ± 3.3^b^
	TYLCV-infected	19	73.7 ± 4.5^a^	69.0 ± 4.1^a^	25.1 ± 2.4^b^
MED	Uninfected	16	76.9 ± 4.6^a^	68.1 ± 5.2^a^	27.0 ± 2.1^b^
	TYLCV-infected	16	63.8 ± 6.1^a,b^	51.9 ± 7.1^b^	39.4 ± 3.8^a^

Live adults and eggs were counted seven days after the newly emerged whitefly adults were released onto the plants to assess adult survival and fecundity.

ªTwo-factor ANOVA: *F*_whitefly_ = 1.001, *df* = 1, p > 0.05; *F*_Plant status_ = 0.128, *df* = 1, p > 0.05; *F*_whitefly*Plant status_ = 6.857, *df* = 1, p < 0.05.

^b^Two-factor ANOVA: *F*_whitefly_ = 0.502, *df* = 1, p > 0.05; *F*_Plant status_ = 0.677, *df* = 1, p > 0.05; *F*_whitefly*Plant status_ = 4.347, *df* = 1, p < 0.05.

^c^Two-factor ANOVA: *F*_whitefly_ = 7.315, *df* = 1, p < 0.05; *F*_Plant status_ = 4.226, *df* = 1, p < 0.05; *F*_whitefly*Plant status_ = 4.547, *df* = 1, p < 0.05.

Different letters in the same column indicate significant differences at P ≤ 0.05 level.

**Table 3:** Performance of MEAM1 and MED whiteflies reared on uninfected and TYLCV-infected plants of tomato *spr2* mutant.

Whitefly	Status of plants	No. of repl.	Survival of females % ± SEª	Survival of males % ± SE^b^	No. of eggs per female ± SE^c^
MEAM1	Uninfected	11	63.9 ± 7.7^c^	66.9 ± 7.3^b^	28.6 ± 2.4^a,b^
	TYLCV-infected	11	83.6 ± 4.1^a,b^	80.0 ± 4.9^a,b^	30.2 ± 3.7^a^
MED	Uninfected	14	75.0 ± 5.2^b,c^	66.4 ± 6.5^b^	23.1 ± 2.0^b^
	TYLCV-infected	13	91.5 ± 3.7^a^	83.9 ± 3.1^a^	15.5 ± 9.1^c^

Live adults and eggs were counted seven days after the newly emerged whitefly adults were released onto the plants to assess adult survival and fecundity.

ªTwo-factor ANOVA: *F*_whitefly_ = 3.093, *df* = 1, p > 0.05; *F*_Plant status_ = 12.611, *df* = 1, p < 0.05; *F*_whitefly*Plant status_ = 0.184, *df* = 1, p > 0.05.

^b^Two-factor ANOVA: *F*_whitefly_ = 0.126, *df* = 1, p > 0.05; *F*_Plant status_ = 7.467, *df* = 1, p < 0.05; *F*_whitefly*Plant status_ = 0.021, *df* = 1, p > 0.05.

^c^Two-factor ANOVA: *F*_whitefly_ = 19.189, *df* = 1, p < 0.001; *F*_Plant status_ = 1.686, *df* = 1, p > 0.05; *F*_whitefly*Plant status_ = 4.086, *df* = 1, p < 0.05.

Different letters in the same column indicate significant differences at P ≤ 0.05 level.

**Table 4:** Performance of MEAM1 and MED whiteflies reared on uninfected and TYLCV-infected transgenic plants of tomato *35S-prosystemin*.

Whitefly	Status of plants	No. of repl.	Survival of females % ± SEª	Survival of males % ± SE^b^	No. of eggs per female ± SE^c^
MEAM1	Uninfected	10	42.0 ± 6.3^b^	63.0 ± 5.2^b,c^	20.7 ± 2.1^b^
	TYLCV-infected	10	77.0 ± 7.3^a^	87.0 ± 3.0^a^	38.4 ± 6.6^a^
MED	Uninfected	11	26.4 ± 4.1^b^	45.5 ± 5.9^c^	19.1 ± 2.0^b^
	TYLCV-infected	11	63.6 ± 9.2^a^	71.8 ± 8.5^a,b^	24.1 ± 3.9^b^

Live adults and eggs were counted seven days after the newly emerged whitefly adults were released onto the plants to assess adult survival and fecundity.

ªTwo-factor ANOVA: *F*_whitefly_ = 3.985, *df* = 1, p > 0.05; *F*_Plant status_ = 23.650, *df* = 1, p < 0.001; *F*_whitefly*Plant status_ = 0.002, *df* = 1, p > 0.05.

^b^Two-factor ANOVA: *F*_whitefly_ = 6.303, *df* = 1, p < 0.05; *F*_Plant status_ = 17.898, *df* = 1, p < 0.001; *F*_whitefly*Plant status_ = 0.001, *df* = 1, p > 0.05.

^c^Two-factor ANOVA: *F*_whitefly_ = 3.942, *df* = 1, p > 0.05; *F*_Plant status_ = 7.946, *df* = 1, p < 0.05; *F*_whitefly*Plant status_ = 2.467, *df* = 1, p > 0.05.

Different letters in the same column indicate significant differences at P ≤ 0.05 level.

### RNA isolation and quantitative real-time PCR (qRT-PCR) analysis

Following our observation that whitefly performance was better on TYLCV-infected *35S-prosystemin* transgenic plants than that on uninfected plants, we compared the expression of JA biosynthesis and defense marker genes in the plants using qRT–PCR. Three replicate plants in each of the two treatments (uninfected and TYLCV-infected) were sampled. Total RNA was isolated using TRIzol Reagent (Invitrogen, Carlsbad, CA, USA). RNA purity and quantity were determined using Nanodrop (Nanodrop Technologies), and 1000 ng of total RNA for each sample was reverse transcribed using the PrimeScript RT reagent Kit with gDNA Eraser (Takara, Japan) according to the manufacturer’s instructions. qRT-PCRs were performed on the Bio-Rad CFX96^TM^ Real-Time System (Bio-Rad, CA, USA) using the SYBR Premix Ex Taq^TM^ II (Takara, Japan). Three technical qRT-PCR replicates were analysed for each biological replicate. The relative expression levels were calculated with the 2^−ΔΔCT^ method and further log_2_-transformed as described by Yuan *et al*.^25^ and Luan *et al*.^26^. The *actin* gene was used as reference. Primers for qRT-PCRs were shown in Table 5.

**Table 5:** DNA primers used in this study.

Gene	Primer sequence
*actin*	Forward primer: TGGTCGGAATGGGACAGAAG
	Reverse primer: CTCAGTCAGGAGAACAGGGT
*AOC*	Forward primer: CAGCAGGACTCTGCATTCTG
	Reverse primer: CGGTGACGGCTAGGTAAGTT
*AOS*	Forward primer: CGATTACCTCCGATTCTGGT
	Reverse primer: AAATCTTCATCCCACCGAAG
*OPR3*	Forward primer: ATGTTGGTCGTGCATCTCAT
	Reverse primer: GGTTCCAATTGCTCTTGGTT
*LOXD*	Forward primer: GGCTTGCTTTACTCCTGGTC
	Reverse primer: AAATCAAAGCGCCAGTTCTT
*PI-II*	Forward primer: TGATGAACCCAAGGCAAATA
	Reverse primer: ACACAACTTGATGCCCACAT

## Data analysis

Statistical significance was evaluated using ANOVA at a 0.05 level followed by LSD tests for whitefly performance. Data of percentages of adult survival were transformed by arcsine square root before analysis. Differences in gene expression levels were analyzed by nonparametric tests. All the data analyses were performed using IBM Statistics SPSS 20.0.

## Results

### Whitefly performance on three types of tomato

The survival of both females and males was not significantly affected by whitefly species or whitefly*plant interaction, but was significantly affected by the types of plants (Table 1). In both species of whiteflies, the percentages of survival were significantly reduced on *35S-prosystemin* compared to those on either *CastleMart* or *spr2*, and in females of MEAM1 survival was significantly higher on *spr2* than that on *CastleMart* (Table 1).

The fecundity was significantly affected by species of whiteflies, types of plants and the interaction between the two factors (Table 1). In MEAM1, fecundity was significantly higher on *spr2* than that on either *CastleMart* or *35S-prosystemin*; while in MED, fecundity was significantly higher on *spr2* but lower on *35S-prosystemin* than that on *CastleMart* (Table 1).

### Whitefly performance on uninfected and TYLCV-infected *CastleMart* plants

On plants of *CastleMart*, the percentages of survival of both females and males were not significantly affected by species of whiteflies or virus-infection status of the plants, but were significantly affected by the interaction between the two factors (Table 2). The survival of MEAM1 females was significantly higher on TYLCV-infected plants than that on uninfected plants; however, the survival of MED females was not significantly affected by the virus-infection status of the plants. The survival of MEAM1 males was not significantly affected by the virus-infection status of the plants, while the survival of MED males was significantly lower on TYLCV-infected plants than that on uninfected plants (Table 2).

The fecundity was significantly affected by species of whiteflies, virus-infection status of the plants, and the interaction between the two factors (Table 2). While the fecundity of MEAM1 did not differ between uninfected and TYLCV-infected plants, the fecundity of MED was significantly higher on TYLCV-infected plants than that on uninfected plants (Table 2).

### Whitefly performance on uninfected and TYLCV-infected *spr2* plants

On plants of *spr2*, the percentages of survival of both females and males were significantly affected by the virus-infection status of the plants, but were not significantly affected by either the species of whiteflies or the interaction between the two factors (Table 3). The survival of females of both MEAM1 and MED was significantly higher on TYLCV-infected plants than that on uninfected plants; the percentages of survival of MEAM1 males did not differ between uninfected and TYLCV-infected plants, while survival of MED males was significantly higher on virus-infected plants than that on uninfected plants (Table 3).

The fecundity was significantly affected by species of whiteflies as well as the interaction between whitefly species and virus-infection status of the plants, but was not significantly affected by the virus-infection status of the plants (Table 3). For MEAM1, fecundity was not significantly affected by the virus-infection status of the plants, while the fecundity of MED was significantly lower on TYLCV-infected than that on uninfected plants (Table 3).

### Whitefly performance on uninfected and TYLCV-infected *35S-prosystemin* plants

On *35S-prosystemin* transgenic plants, the percentages of survival of females were not significantly affected by species of whiteflies or the interaction between whitefly species and virus-infection status of the plants, but were significantly affected by the virus-infection status of the plants. In both whitefly species, survival of females was significantly higher on TYLCV-infected plants than that on uninfected plants (Table 4). The percentages of survival of males were significantly affected by species of whiteflies and the virus-infection status of the plants, but were not affected by the interaction between the two factors. In both whitefly species, survival of males was also significantly higher on TYLCV-infected than that on uninfected plants (Table 4).

The fecundity was significantly affected by the virus-infection status of the plants, but was not significantly affected by the species of whiteflies or the interaction between the two factors. While the fecundity of MEAM1 was significantly higher on TYLCV-infected plants than that on uninfected plants, the fecundity of MED did not differ between uninfected and TYLCV-infected plants (Table 4).

### Expression of JA signaling pathway-related genes in uninfected and TYLCV-infected plants of *35S-prosystemin*

**Figure 1:**
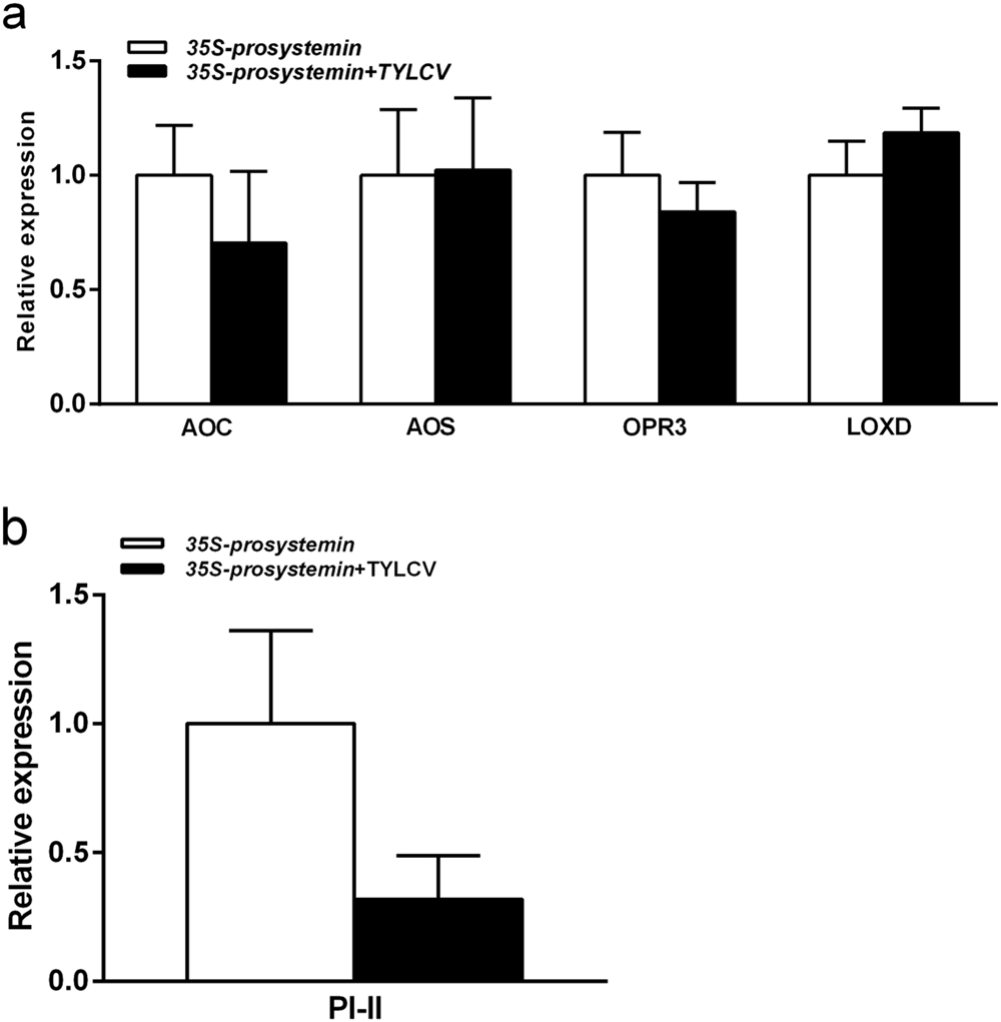
The transcript levels of JA pathway-related genes in uninfected and TYLCV-infected *35S-prosystemin* transgenic tomato plants. (**a**) Relative expression level of biosynthesis genes in JA signaling pathway; and (**b**) PI-II at downstream of JA pathway. The error bars represent SEM.

The transcript levels of all four JA-biosynthesis-related genes we tested did not differ significantly between uninfected and TYLCV-infected *35S-prosystemin* plants (Fig. 1a); however, the expression of the *PI-II* gene at the downstream of JA pathway was marginally repressed in TYLCV-infected plants compared to that in uninfected plants (*P* = 0.061; Fig. 1b).

## Discussion

JA signaling pathway is involved extensively in plant resistance against herbivore insects^27, 28^. JA signaling pathway regulates the production of many plant secondary metabolites and defensive proteins that are harmful to herbivores, for example, terpenoids in tobacco plant play an important role in the resistance against whiteflies^3, 29–32^. Our data shows that, in tomato, the two species of whiteflies performed poorer on plants exhibiting a higher level of JA-related resistance (Table 1). TYLCV-infection of tomato plants repressed their JA-related resistance (Fig. 1), and TYLCV caused the infected plants to promote the performance of the whiteflies (Table 4). Further, this effect of promotion of whitefly performance by TYLCV infection was stronger on plants with a higher level of JA-related resistance than on plant with a lower level of JA-related resistance (Tables 2–4).

During the experiments, the plants of the *CastleMart* wild type, and JA-deficient *spr2* mutant and JA-overexpression *35S-prosystemin* transgenic plants grew to a similar size but did appear somewhat different. The differences among them are difficult to describe. In the experiments, test adults were caged on leaves of tomato plants, with the same number of adults enclosed in the same type of clip-cage. So the average area of leaf per insect was the same for all replicates in each of the treatments. We assumed that any differences in the phenotype of the plants had little effect on the performance of the whiteflies when we were comparing the performance of whiteflies in different treatments.

JA-induced defenses in *Arabidopsis thaliana* was shown to confer resistance to whiteflies^33^. Previous studies have demonstrated that the begomovirus Tomato Yellow leaf curl China virus (TYLCCNV) can promote the performance of whiteflies on infected tobacco plants through inhibiting JA-related defence^32, 34^. The pathogenicity factor ßC1 of TYLCCNV was found to be interacting with MYC2, and the interactions drive the down-regulation of JA signaling pathway and in turn suppression of terpenoid synthesis and release^30^. However, in this study, the begomovirus TYLCV infection did not inhibit the expression of the four JA-biosynthesis genes (*AOC, AOS, OPR3, LOXD*) in the *35S-prosystemin* transgenic plants, but the transcript level of the JA-regulated defense gene (*PI-II*) was down-regulated (Fig. 1). It seemed likely that TYLCV caused the infected tomato plants to promote whitefly performance not via a direct repression of the JA synthesis pathway.

In this study, two species of whiteflies of the *B. tabaci* complex were included in the experiments. Statistical analyses of the data indicated that in some cases whitefly species had a significant role in the interactions (Tables 1–4). For example, on plants of the wild type, TYLCV-infection of the plants appeared to have some positive effects on the survival of MEAM1 females but negative effects on the survival of MED males. Further, TYLCV-infection of the plants did not have an effect on the fecundity of MEAM1 but a significant positive effect on the fecundity of MED. The mechanisms under these differences are not yet known. Similarly, in a previous study, we showed that both TYLCV and TYLCCNV caused the infected tomato plants of a widely grown cultivar Hezuo903 to promote the performance of the invasive MEAM1 but repressed the performance of an indigenous species of whitefly Asia II 3^21^. In another study, we found that TYLCV infection of tomato plants had little effects on the performance of both the invasive MED and the indigenous Asia II 1 whiteflies^20^. These results are not surprising but do help to show the complexity that can occur in the tripartite interactions between whiteflies, begomoviruses and host plants. The data of this study indicate that the effects of virus infection of the plants on the performance of whiteflies vary with the intrinsic level of JA-related resistance (Tables 1–4), with plants that have higher intrinsic JA-related defence/resistance showing stronger positive effects on whitefly performance. Tomato plants are in general highly suitable to whitefly infestation possibly due to, in part, a low level of intrinsic JA-related defence/resistance. This inference may provide some clues to understand that in many cases viral infection of tomato plants does not have a major role in affecting whitefly performance on these highly suitable plants^18–20, 35^.

All in all, we show that begomovirus infection may suppresses JA-related resistance in tomato plants and in turn promote the performance of whiteflies. However, this benefit effect conferred to the whiteflies via infection of the host plants by the virus becomes significant or substantial only when JA-related resistance plays a major role in the plants against whiteflies. Our findings confirmed the general role of JA-related resistance against whiteflies, and further show that the effects of suppression of JA-related resistance in plants by begomoviruses on whitefly performance may vary depending on the intrinsic role of JA in that plant.
